# Anticancer Metallocenes and Metal Complexes of Transition Elements from Groups 4 to 7

**DOI:** 10.3390/molecules29040824

**Published:** 2024-02-11

**Authors:** Irena Kostova

**Affiliations:** Department of Chemistry, Faculty of Pharmacy, Medical University-Sofia, 1000 Sofia, Bulgaria; irenakostova@yahoo.com or i.kostova@pharmfac.mu-sofia.bg

**Keywords:** transition metals, metallocenes, coordination complexes, drug design, cancer diagnostics and therapy

## Abstract

With the progression in the field of bioinorganic chemistry, the role of transition metal complexes as the most widely used therapeutics is becoming a more and more attractive research area. The complexes of transition metals possess a great variety of attractive pharmacological properties, including anticancer, anti-inflammatory, antioxidant, anti-infective, etc., activities. Transition metal complexes have proven to be potential alternatives to biologically active organic compounds, especially as antitumor agents. The performance of metal coordination compounds in living systems is anticipated to differ generally from the action of non-metal-containing drugs and may offer unique diagnostic and/or therapeutic opportunities. In this review, the rapid development and application of metallocenes and metal complexes of elements from Groups 4 to 7 in cancer diagnostics and therapy have been summarized. Most of the heavy metals discussed in the current review are newly discovered metals. That is why the use of their metal-based compounds has attracted a lot of attention concerning their organometallic and coordination chemistry. All of this imposes more systematic studies on their biological activity, biocompatibility, and toxicity and presupposes further investigations.

## 1. Introduction

Since the discovery of the first metal-based compounds with proven anticancer activity, great efforts have focused on the rational design of new metal complexes which can potentially be used in cancer chemotherapy. In the last several decades, a great number of metal coordination compounds have been extensively studied and evaluated in vitro and in vivo as potent antineoplastic agents, some of them being at different stages of clinical trials. The main representatives from the studied d-block transition metals are platinum [1], ruthenium [2], rhodium [3], palladium [4], iridium [5], gold [6], silver [7], etc., and their complexes. Many of these antineoplastic agents have been intensively addressed in the issued literature [8,9,10] and are beyond the scope of the present review.

d-Elements are trace microelements with different oxidation states and a high affinity to various donors of biomolecules. They are essential in various cellular functions, being involved in most vital life processes such as enzymatic, synthetic, electron, and ion transfer reactions, as well as energy processes. Transition metal cations are chemically very reactive and participate in numerous biochemical interactions with biomolecules in living systems which leads to the formation of different metal-based scaffolds in vivo. Due to their partially filled d-shells, d-elements are predisposed to complexation reactions. The variable number of electrons in the d-shell impacts the magnetic and electronic properties of the resulting metal complexes.

Transition metal complexes can adopt various geometries depending on the number of coordination bonds they possess, for instance octahedral, square-planar, and square- and trigonal-bipyramidal, whereas organic compounds are only limited to tetrahedral, planar, and linear geometries, which gives rise to some structural diversity of metal-based compounds. This characteristic improves the flexibility in drug design of metal complexes, their thermodynamic and kinetic properties, and the opportunity to effectively interact with the target biomolecules. Metal cations possess individual features and unique biochemical properties such as charge variation, Lewis acid properties, flexible coordination modes and a wide range of geometries, different oxidation states and redox activity, metal–ligand interactions, and exchange reactions. All of these attractive characteristics determine their great therapeutic potential. Moreover, the therapeutic mechanisms of action of metal-based compounds are completely different than those of pure organic compounds.

In the past several decades, a number of classes of new metal-based coordination compounds have been explored as potent anticancer agents based on a wide variety of d-elements and their properties and diverse modes of action. Because of the excellent review coverage on the chemistry, biology, and medicine of platinum (Pt^2+^ and Pt^4+^), ruthenium (Ru^2+^ and Ru^3+^), gold (Au^+^ and Au^3+^), and silver (Ag^+^) anticancer drugs [1,2,3,4,5,6,7,8,9,10], this review is concentrated on some of the most recent advances regarding the metallocenes and metal complexes of d-elements from Groups 4 to 7 of the periodic table. The review is focused on the promising results obtained with monometallic complexes of these groups along with the new approach of combining different metal centers into heterometallic complexes which allows combinations of various mechanisms of action and possible synergistic effects. 

The d-elements from Groups 4 to 7 possess different chemical and pharmacological properties and diverse modes of action which can modify pathways in cellular metabolism or adjust some chemical properties and biofunctions of the obtained complexes, such as the solubility, lipophilicity, etc. The biological functions, medical applications related to their anticancer activity, and toxic effects of the most studied elements from Groups 4 to 7 and their compounds are collected in Table 1.

## 2. d-Elements of Group 4

### 2.1. Titanocenes and Other Ti(IV) Complexes

**Figure 1:**
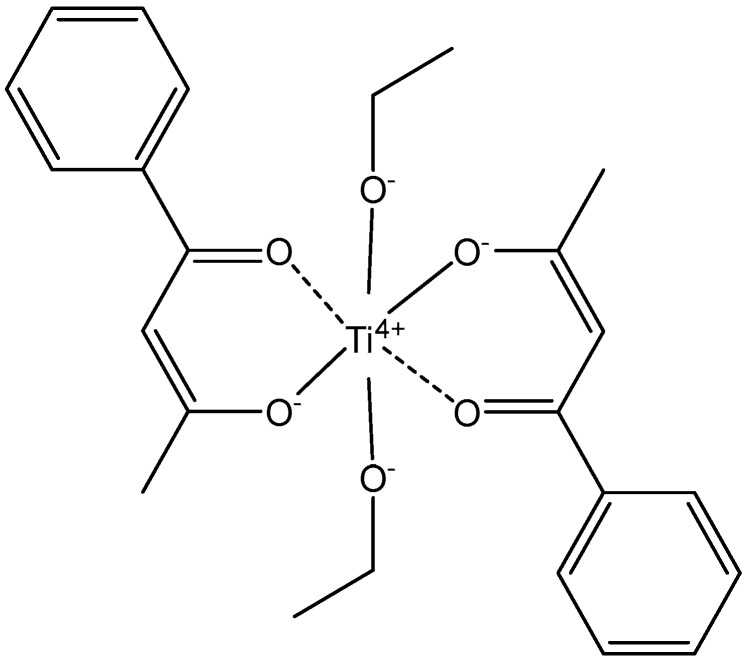
The structure of budotitane.

Titanium(IV) complexes were one of the first classes of metal complexes to enter clinical trials after Pt anticancer complexes with a distinct mechanism of action and spectrum of activity from Pt(II) and Pt(IV) complexes [56]. That is why they are recommended to be considered as potential candidates in the treatment of cases of resistance to cisplatin. In contrast to the platinum complexes, titanium derivatives showed no indication of nephrotoxicity or myelotoxicity. The first metallocene revealed to have antitumor activity was titanocene dichloride. Budotitane (Figure 1) and titanocene dichloride exhibit antitumor activity with little toxicity in many tumor cells. TiCp_2_Cl_2_ and budotitane ([Ti(IV)(bzac)_2_(OEt)_2_], where bzac = 1-phenylbutane-1,3-diketonate) were the first Ti(IV) compounds to enter clinical trials, but finally failed because of their nonsufficient water stability and unspecified mechanism of action. TiCp_2_Cl_2_ and budotitane were the first antitumor metal complexes to enter clinical trials after Pt compounds [57]. With their two labile ligands, these complexes hydrolyze rapidly in water solutions. Nevertheless, their active species and action mechanism continue to be unclear and undefined. It has been found that in the series cis-[M(IV)(bzac)_2_X_2_], analogues to budotitane, the activity varied in the order Ti ≈ Zr > Hf > Mo > Sn > Ge, which is roughly inverse to the rates of X dissociating from the metal ion. Little is known about the mechanism of action of budotitane, except that it is different from that of cisplatin, with no significant DNA damage. Most probably the mechanism of budotitane bears analogy to those of TiCp_2_Cl_2_ and some active Ru complexes.

The cis-dichloride structure of TiCp_2_Cl_2_ makes interesting parallels with cisplatin, but it hydrolyzes in water to various species and it is not clear which is the active one. The main disadvantage of Ti(IV) complexes is their low stability in aqueous solutions. The current investigations are mainly focused on water-soluble and stable Ti(IV) antineoplastic complexes. Thereafter, new Ti(IV) complexes have been designed to overcome the low stability. Between them, substituted titanocenes and titanium salan compounds have shown potential that might offer a greater stability, water solubility, and cytotoxicity [58]. Titanocene-Y contains methoxyphenyl substituents of the Cp rings which give a higher potency. The Ti complexes Ti–salan and titanocene-Y exhibit contrasting actions related to their interactions with DNA and albumin, cellular uptake, and intracellular circulation. Salan-based complexes are a well-established class of coordination complexes. Ti–salan demonstrates relatively low binding to biomolecules but increased serum-dependent cellular uptake, while titanocene-Y shows lower cellular accumulation and high binding to albumin and DNA. The biodistribution data have specified that for titanocene-Y the DNA reactions are critical, whereas for Ti–salan mitochondrial targeting is significant. The introduction of 2,6-dipicolinic acid as a second chelator to Ti–salan has resulted in novel heteroleptic complexes with a good aqueous stability and remarkable in vitro and in vivo cytotoxicity [59]. This ligand system has demonstrated a high adoptability to Group 4 elements. A series of C1 symmetrical Ti(IV)–salan complexes of differently substituted -NO_2_, -Cl, and -Br aromatic ligands, halogenated on one ring and nitrated on the other, have been reported to demonstrate high antineoplastic activity, combining the higher activity and stability with enhanced solubility [60]. These hybrid complexes are highly stable and have been found to possess much higher antitumor activity than C2 symmetrical Ti(IV)–salan analogues with the same substituents and cisplatin. A particular advantage of the C1 symmetrical complexes is their improved solubility in DMSO, which is important for therapeutic applications. The same authors have continued their study to hydrolytically stable trans-Ti(IV) complexes containing the salophen ligand [60]. The trans-Ti(IV) complexes exhibited high stability and good cytotoxicity against different cancer cell lines. 

**Figure 2:**
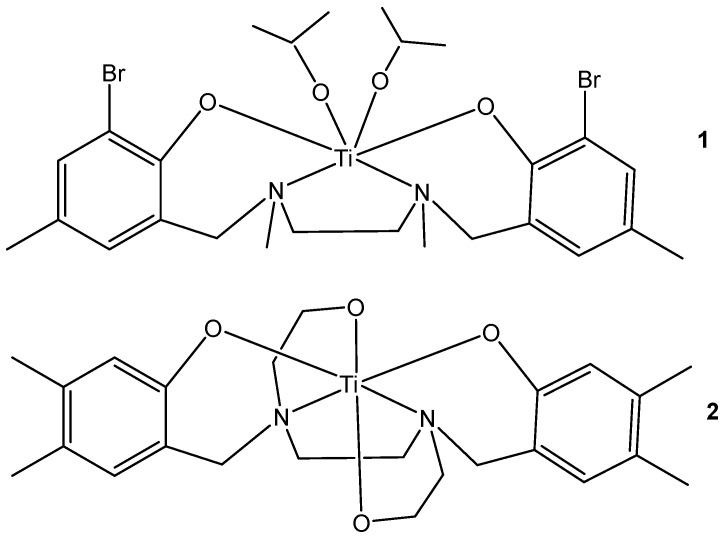
Structures of Ti(IV)–salan (1) and PhenolaTi (2).

The promising diaminobis(phenolato)bis(alkoxo) Ti(IV) anticancer complex (PhenolaTi) has shown remarkable cytotoxicity with no detected toxicity in animals [61]. The structures of Ti(IV)–salan and PhenolaTi are presented in Figure 2.

PhenolaTi has induced apoptosis and cell-cycle arrest at the G2/M phase in MCF7 cells. It has been shown that, unlike cisplatin and related known metallodrugs, no inhibition of DNA polymerase activity was detected, which means a distinct ER-related mechanism by this Ti(IV) complex. The precise interactions between PhenolaTi and its targets are yet to be clarified, along with the reason for tumor selectivity and non-toxic effects for this Ti(IV) complex [62].

Novel antitumor diamino-bis-(phenolato) [ONON] type titanium(IV) complexes stabilized by 2,6-dipicolinic acid have recently been synthesized [63]. The complexes have displayed improved inhibiting activity against Hep G2 cells compared to salan–Ti(IV) complexes. 

The success of cisplatin and TiCp_2_Cl_2_ has stimulated many scientific groups to search for similar metal complexes containing reactive Cl^−^ anions in the *cis*-position with vanadium, chromium, niobium, molybdenum, manganese, and their analogs. The metallocene dihalides MCp_2_X_2_, where Cp = η^5^-cyclopentadienide and X = halide, represent a class of small, hydrophobic anticancer candidates. They undergo rapid hydrolysis in water solutions [58]. These compounds have distorted tetrahedral structures where two cyclopentadienyl ligands and two halide- or acido-ligands (X) are coordinated to the metal in the +4 oxidation state. The two Cp rings with delocalized negative charges are bonded to the metal center in a bent sandwich configuration. Complexes of MCp_2_Cl_2_ exhibit activity against many tumor cells such as the leukemias P388 and L1210, B16 melanoma, colon, and Lewis lung carcinomas, solid and fluid Ehrlich ascites tumors, and several human colon and lung carcinomas [58]. 

The antineoplastic activity of metallocenes of MCp_2_X_2_ depends on the metal. The complexes with Ti, V, Nb, and Mo are active, but the complexes with Ta and W show insignificant activity, and these with Zr and Hf are inactive. Titanocene and vanadocene dichlorides have exhibited the best action against lung, breast, and gastrointestinal cancers in vivo. Variations in halide and diacido ions have been widely studied mainly for titanocene dichloride. It has been found that halides do not affect the antitumor activity. Fractional studies have been performed on different substituents in Cp ligand structures, and almost all of them have been limited to the titanocenes [56]. 

The dose-limiting toxic effects of TiCp_2_Cl_2_ include nephrotoxicity and elevation of creatinine and bilirubin levels, which are cumulative but reversible. The detected hepatotoxicity and gastrointestinal toxicity for titanocene dichloride are also the main disadvantages. Concerning the mechanism of action, it is still not clear whether DNA is the main target for Ti(IV) ions. The binding of titanium to nitrogen atoms in DNA appears to be weak at neutral pH. It is supposed that the phosphate groups are the preferred ligands for titanium. The binding of Ti(IV) to the Fe(III)-transport protein transferrin may also play a significant role [56,58]. Furthermore, the studies of the hydrolysis processes, stability at different pH, and the interactions with nucleic acids have established that each of the metallocene representatives has its own mechanism of action specific for the respective metal cation. The metallocenes of MCp_2_X_2_ have been initially tested as possible analogues of cisplatin, but the effectiveness of TiCp_2_Cl_2_ against Pt-resistant cell lines indicated a completely different mechanism of action that may lead to new therapeutic options against some types of cancer. The antitumor activity of the metallocenes of MCp_2_X_2_ probably depends on the hydrolysis of the metal, which for Ti, V, Zr, and Mo proceeds much faster than for cisplatin. Some studies have suggested a correlation between DNA binding and anticancer activity in that immediate complexation of the DNA nucleotides occurred with the active Ti and Mo metallocenes, whereas the biologically inactive Hf and Zr metallocenes did not bind significantly to DNA [64].

**Figure 3:**
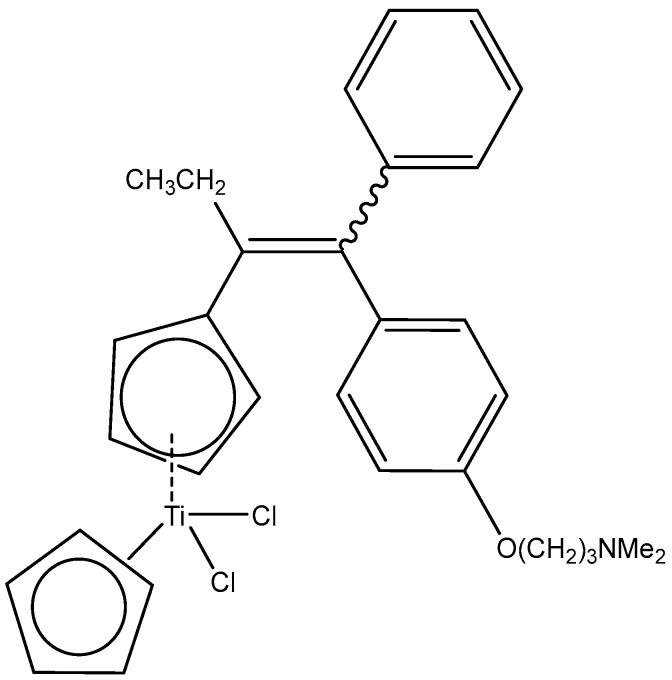
The structure of a titanocene derivative of the anticancer drug tamoxifen.

The titanocene derivative of tamoxifen (Figure 3) with antineoplastic activity revealed a superior proliferative action on the estrogen-dependent tumor cells MCF7, derived from a breast cancer line holding ER+ [56], similar to that detected with TiCp_2_Cl_2_. It has to be mentioned that the resistance to tamoxifen, found in many types of breast cancer, remains the most significant problem. 

Numerous second- and third-generation antitumor titanium complexes have been developed in order to reduce the decomposition rates and to increase the cellular uptake and cytotoxic activity [65,66]. The most promising candidates possess an increased lipophilicity of cyclopentadiene ligands, for instance, titanocene Y [67], complexes of hexadentate ligands with increased hydrolytic stability [68] and complexes possessing resistance to transferrin binding [69]. 

Recently, a series of titanocene(IV) carboxylate complexes have been synthesized, which were more resistant to hydrolysis than titanocene dichloride. The newly synthesized titanocene dicarboxylates have been tested on MCF7 and MCF7-10A cells [70]. Provoked by the challenge to develop soluble and bio-obtainable forms of Ti(IV) compounds, new binary Ti(IV)-(α-hydroxycarboxylic acid) complexes, incorporating natural ligands (α-hydroxy isobutyric acid, D-quinic acid, 2-ethyl-2-hydroxybutyric acid) have been isolated [71]. The cytotoxicity studies in 3T3-L1, Saos-2, and KS483 cell lines led to the formulation of a well-defined antiproliferative profile for the tested compounds.

**Figure 4:**
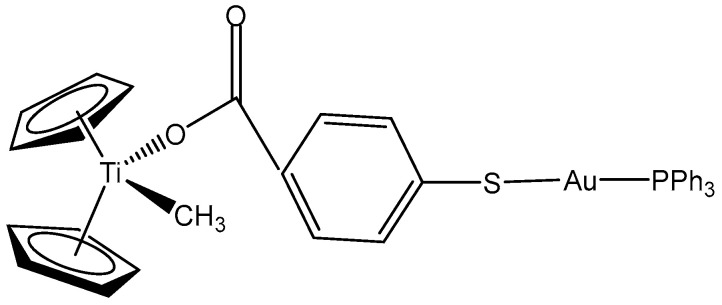
Titanocene complex [(η-C_5_H_5_)_2_TiMe(μ-mba)Au(PPh_3_)].

The inclusion of additional transition metal ions with different properties can functionally complement parent monometallic complexes. This strategy has been employed to develop new heterometallic compounds as antitumor therapeutics [72]. Due to the relatively low stability of the Cp-Ti bond, another metal (Au) has been incorporated to the benzoate ligand in order to increase the stability of the parent compound. The synthesis, characterization, and stability studies of new titanocene complexes containing a methyl group and a carboxylate ligand (mba = S–C_6_H_4_–COO^−^) bound to gold(I)-phosphane fragments through a thiolate group have been reported. The obtained titanocene complex [(η-C_5_H_5_)_2_TiMe(μ-mba)Au(PPh_3_)] (Figure 4) has been identified as the most active, showing high cytotoxicity to Caki-1 renal cancer cell line by inducing apoptosis and necrosis. Preliminary mechanistic studies in Caki-1 renal cells have indicated that the cytotoxic and anti-migration effects of [(η-C_5_H_5_)_2_TiMe(μ-mba)Au(PPh_3_)] involve the inhibition of thioredoxin reductase and loss of expression of protein kinases that drive cell migration (AKT, p90-RSK, and MAPKAPK3). The colocalization of both titanium and gold metals in a ratio of 1:1 in Caki-1 renal cells has been shown to indicate the strength of the heterometallic compound in vitro. In a Caki-1 renal cancer xenograft mice model, the compound has demonstrated only minor toxic effects. 

**Figure 5:**
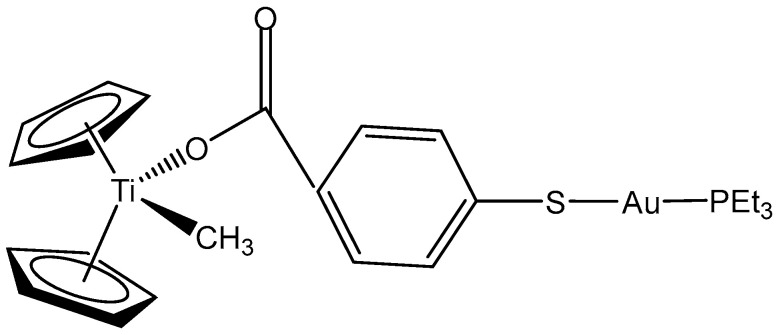
Titanocene complex [(η-C_5_H_5_)_2_TiMe(μ-mba)Au(PEt_3_)].

The same group has another heterobimetallic Ti(IV)-Au(I) complex with a different substituent [(η-C_5_H_5_)_2_TiMe(μ-mba)Au(PEt_3_)] (Figure 5). 

The mechanism of action of Ti(IV)-Au(I) complexes was different compared to the classical drug cisplatin. These complexes did not bind DNA, but they inhibited protein kinases such as p90-RSK, AKT, MAPKAPA, and thioredoxin reductase [73]. The two new Ti(IV)-Au(I) complexes have shown IC_50_ values under the µM range in the case of the renal cancer cell line Caki-1 after 72 h incubation. These bimetallic compounds have been found to be more cytotoxic than the Au(I) monometallic derivatives and much more cytotoxic than the titanocene dichloride, with effective antimigration and anti-invasive properties, making them promising candidates for cancer chemotherapy.

### 2.2. Zirconium(IV) Complexes

In aqueous solutions, Zr exists mainly in the +IV oxidation state, although some other oxidation states (+I, +II, +III) have also been described. The Zr(IV) ion, with its [Kr]4d^0^ electronic configuration, is a highly charged cation with a small ionic size, which can form complexes with flexible coordination geometries with variable coordination numbers from 4 to 12, the most common being 6 and 8. The Zr^+4^ ion with its high charge and small radius is a typical hard Lewis acid, having a strong affinity for hard Lewis bases with O or N donor atoms, and very sporadically with S donor atoms. 

Zirconium possesses high coordination numbers and the ability to form stable complexes. Despite the success of Ti(IV) complexes, reports for heavier analogues in zirconium(IV) complexes with antitumor activity are rather scarce, which is principally due to the unsatisfactory water stability of Zr(IV) complexes as demonstrated by earlier reports of zirconocene dichloride and its amino derivatives [74] and Zr(IV) 1,3-diketonates [75]. Zr(IV) bis-chelated complexes of salan have demonstrated comparable antitumor activity to cisplatin and good aqueous stability, but they had a low water solubility [76]. 

**Figure 6:**
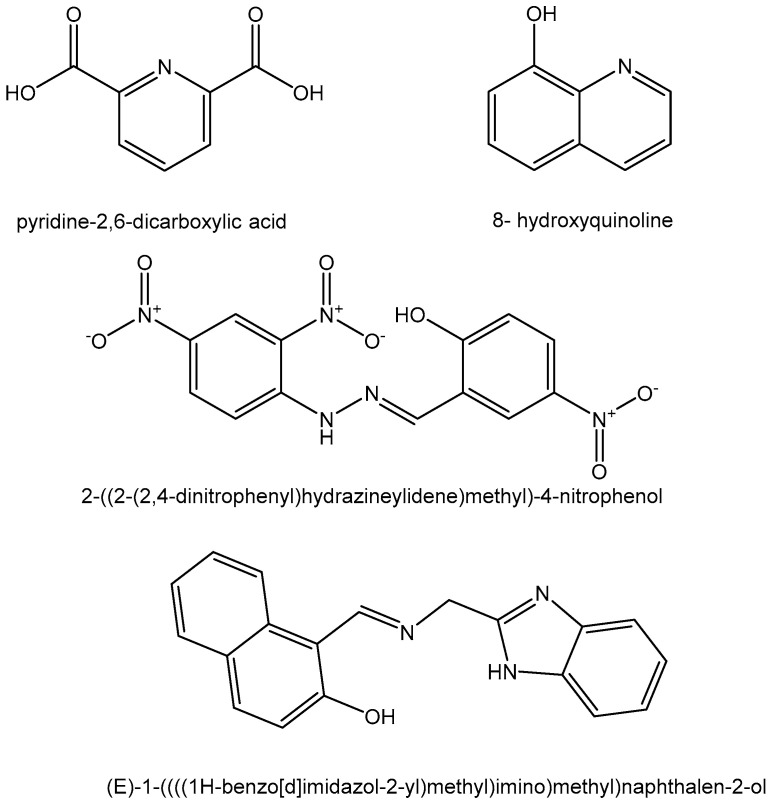
Ligands for zirconium(IV) complexes.

Metallocene-diacido complexes containing titanium, vanadium, niobium, zirconium, and molybdenum have been found to exhibit variable antitumor activity for a wide spectrum of tumors with reduced toxicity as compared to the classical drug cisplatin. Zr(IV) coordination complexes of proton-transfer compounds containing pyridine-2,6-dicarboxylic acid (Figure 6) with 2-methylimidazole and imidazole [2-mimH]_2_[Zr(pydc)_3_] and [imiH]_2_[Zr(pydc)_3_].4H_2_O have been reported. The antiproliferative activity of the tested compounds has been evaluated in vitro against human breast cancer MCF7, human lymphocyte HL60, and human colon adenocarcinoma HT29 cell lines. A significant cytotoxic effect has been observed on MCF7 cells (IC_50_ = 10 μM) [77].

Zirconium(IV) complexes of substituted pyrazole derivatives, e.g., 4-[2-vinylthiophene]-3-methyl pyrozolin-5(4H)-one, 4-[4-chloro benzylidine]-3-methyl pyrozolin-5(4H)-one, and 4-[4-dimethylnitro benzylidine]-3-methylpyrozolin-5(4H)-one, have been synthesized. The tested compounds exhibited considerable antitumor activity and cytotoxic specificity towards a human colon carcinoma HCT-116 cell line [78].

The synthesis and characterization of mixed ligand Zr(IV) complexes of 8-hydroxyquinoline (Figure 6) as a primary ligand and amino acids (L-alanine, L-serine, glycine) as a secondary ligand have been reported [79]. The ligand 8-hydroxyquinoline stabilizes Group 4 (Ti, Zr, Hf) complexes. Zirconium complexes have been screened for their cytotoxic properties on *Ehrlich ascites* and *Daltonís lymphoma ascites* tumor cell lines. New stabilized Zr(IV) complexes of 8-hydroxyquinoline with solid state structures have recently been obtained [80]. The compounds have demonstrated a good solubility and stability in H_2_O and DMSO, which can explain their exceptional inhibition effects against human cervical tumor Hela S3, human-derived hepatoma Hep G2, and human lung cancer PC9 cell lines via an almost entirely induced apoptotic pathway. 

The synthesis, characterization, and reactivity of mononuclear oxy-vanadium(IV) and oxy-zirconium(IV) complexes (VO(ALz)_2_ and ZrO(ALz)_2_) of O,N-monobasic bidenate arylhydazone derivative 2-((2-(2,4-dinitrophenyl)hydrazineylidene)methyl)-4-nitrophenol, HALz (Figure 6) have recently been reported [81]. The biological activity of the complexes was studied within the binding action towards ctDNA. Both V(IV) and Zr(IV) ions in the complexes promoted their reactivity as antioxidant and antineoplastic reagents more than the free ligand. The compounds have been tested against human colon carcinoma HCT-116, human breast adenocarcinoma MCF7, and human hepatocellular carcinoma HepG2 cell lines in vitro. It has been found that the prominent cytotoxic activity of V(IV) and Zr(IV) complexes was due to the attendance of the high valent metal ions. Additionally, the higher Lewis acidity of the V(IV) ion compared to the Zr(IV) ion resulted in more effective biological activity.

Zirconium(IV) and other transition metal Schiff base complexes of (E)-1-((((1H-benzo[d]imidazol-2-yl)methyl)imino)methyl)naphthalen-2-ol (Figure 6) have been synthesized [82]. The in vitro antitumor activities of the complexes were tested against human breast adenocarcinoma MCF-7, human hepatocellular carcinoma HepG2, and human colon carcinoma HCT-116 cells. The zirconium(IV) complex has shown significant effects on the HCT-116 cell line.

**Figure 7:**
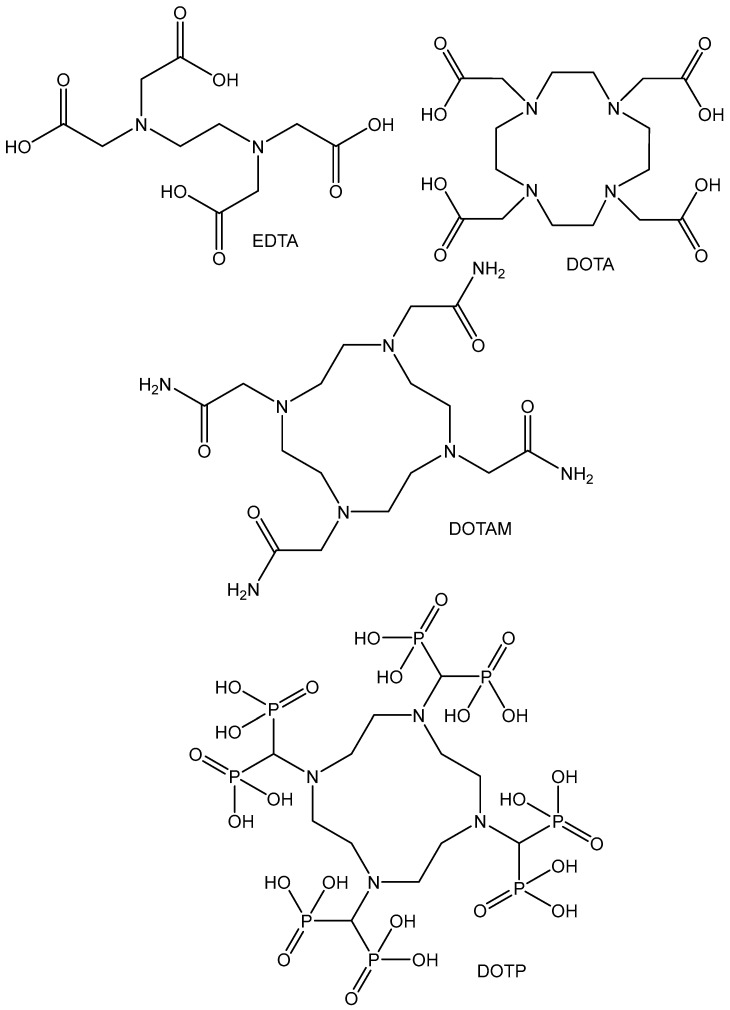
Structures of acyclic (EDTA) and cyclic (DOTA, DOTAM, DOTP) ^89^Zr polyazacarboxylate chelators, used in radiopharmaceutical applications.

Although several isotopes of Zr including ^86^Zr (T_1/2_ = 17 h, γ), ^88^Zr (T_1/2_ = 85 d, γ), and ^89^Zr (T_1/2_ = 78.4 h, β^+^) can be produced, ^89^Zr has received the most attention for radiopharmaceutical applications. Of all the commercially available pure positron emission tomography (PET) radionuclides, ^89^Zr is the only one with a sufficient half-life (T_1/2_ = 3.27 d) to be useful for the labeling of antibodies, antibody fragments, cells, and nanoparticles. The radionuclide zirconium-89 has found extensive use for positron emission tomography imaging when it is coupled with proteins, antibodies, nanoparticles, etc., [83]. Stable coordination of radioactive ^89^Zr^4+^ in an aqueous environment is of critical importance to attach the ^89^Zr radioisotope to the targeting biomolecule. Many papers focused on the design of high-affinity Zr chelators have been reported [84]. Polyazacarboxylate chelators are the best known. Some examples of the structures of acyclic ethylenediaminetetraacetic acid (EDTA) and cyclic (2,2′,2″,2′′′-(1,4,7,10-tetraazacyclododecane-1,4,7,10-tetrayl)tetraacetic acid DOTA, 1,4,7,10-tetrakis(carbamoylmethyl)-1,4,7,10-tetraazacyclododecane DOTAM, 1,4,7,10-tetraazacyclododecane-1,4,7,10-tetra(methylene phosphonic acid) DOTP) ^89^Zr polyazacarboxylate chelators, used in radiopharmaceutical applications, are shown in Figure 7. 

**Figure 8:**
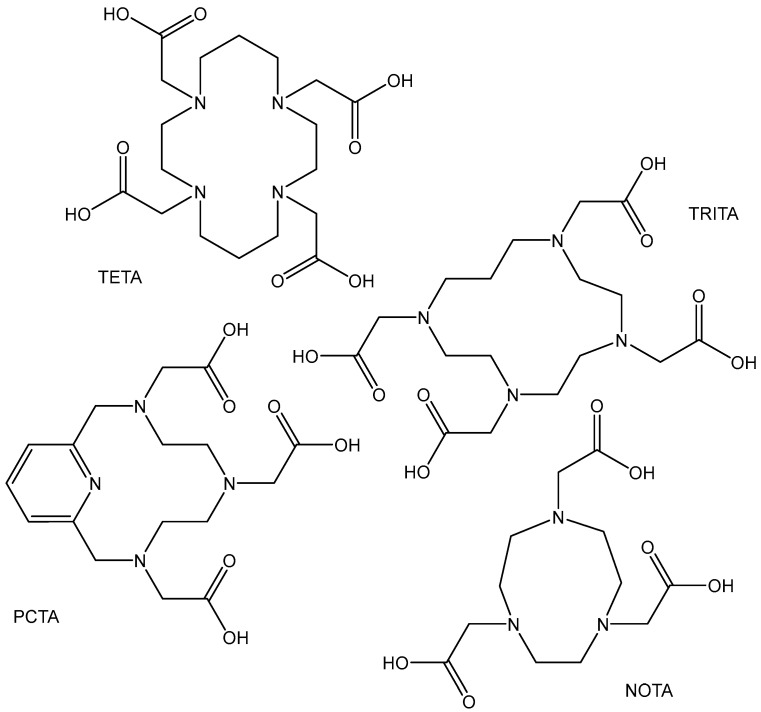
Structures of acyclic ^89^Zr polyazacarboxylate chelators (TETA, TRITA, PCTA, NOTA).

The Zr(IV) cation prefers polyanionic hard donor ligands, as proved by the stability constants of its complexes with the acyclic polyazacarboxylate chelators with a dodecahedron arrangement and a coordination number of eight. Tetraazamacrocycles, like DOTA and its derivatives (DOTAM and DOTP), do not fit well with the high oxophilic demand of the hard Zr(IV) cation. Recent studies have proved the ability of DOTA and its derivatives with phosphonate pendant groups (DOTP) and with hydroxamate pendant groups (DOTAM) to yield stable 8^9^Zr-labeled compounds with octahedral geometry and a good performance in vitro and in vivo [85]. The availability of ^89^Zr oxalate or zirconium-89 chloride is crucial to the development of efficient immuno-PET agents. Radioactive analogs, based on the ^89^Zr-oxalate precursor, resulted in low radiochemical yields, possibly due to the competition between the macrocycle chelator and the oxalates in solution. In contrast, the usage of the zirconium-89 chloride ^89^ZrCl_4_ allowed measurable complexation of the chelators. The studies on the stability of these DOTA-based complexes have confirmed their notable stability. It has been found that the order of the complexes’ stability is ^89^Zr-DOTA > ^89^Zr-DOTP > ^89^Zr-DOTAM. Recently, the same authors have described the synthesis and characterization of Zr complexes with octacoordinated polyazamacrocycle chelators: 1,4,8,11-tetraazacyclotetradecane-1,4, 8,11-tetraacetic acid (TETA), 2,2′,2″,2′′′-(1,4,7,10-tetraazacyclotridecane-1,4,7,10-tetrayl) tetraacetic acid (TRITA), 3,6,9,15-tetraazabicyclo[9.3.1]pentadeca-1(15), 11,13-triene-3,6,9-triacetic acid (PCTA), and 2,2′,2″-(triazacyclononane-1,4,7-triyl)-triacetic acid (NOTA), Figure 8 [86].

The research of the important PET isotope ^89^Zr has developed quickly for understanding zirconium chemistry and for designing ligands that stably chelate ^89^Zr. Many ligands containing hydroxamate, hydroxyisopthalamide, terepthalamide, tetraazamacrocycles, and hydroxypiridinoate coordinating units have been examined to efficiently chelate ^89^Zr.

### 2.3. Hafnium(IV) Complexes

Hafnium is a chemically reactive metal in the titanium-triad of the periodic table, closely related to titanium and zirconium. The ionic radius of Hf(IV) is higher than that of Ti(IV) and similar to that of Zr(IV), which may result in some specific characteristics for the complexes of Hf(IV), for instance, Hf(IV) is a softer Lewis acid than Ti(IV) and Zr(IV) ions. Hafnium coordinates with ligands holding O, N, and S donor atoms in the +4 oxidation state with a preference for soft bases containing nitrogen atoms.

Various Hf(IV) complexes have found different applications, though there are not many reports on their antitumor activity. Cytotoxic Hf(IV) complexes are mainly complexes of β-diketonates, but they have a low stability and undefined mechanism of action. Zhao et al. have recently reported the synthesis of novel heptacoordinated Hf(IV) complexes of salan derivatives and 2,6-dipicolinic acid showing a rapid cellular uptake process [87]. The complexes have been evaluated against the human cervical carcinoma Hela S3 and human-derived hepatoma Hep G2 cells. Nevertheless, their anticancer activity, hydrolytic performance, and the involved interactions remained unclear. Salan Hf(IV) alkoxyl and bimetallic oxidobridged Hf(IV) complexes have been recently obtained [88]. The complexes have demonstrated improved hydrolytic stability and antineoplastic activity against Hela S3 and Hep G2 cell lines. Principally, the hafnium(IV) and zirconium(IV) complexes are very similar with regard to their geometry and coordination modes. Hafnocene dichloride showed no activity against several tumor cell lines, for instance bis- or tris-β-diketonate Hf(IV) complexes had comparable inhibitory activity against the human colon cancer HT-29 and human breast cancer MCF-7 cells compared to cisplatin. In fact, Hf exhibits biomedical advantages such as biocompatibility and low toxicity [89]. Hafnocene dichloride HfO_2_, possessing remarkable chemical inertness, has been used in microneedles for transdermal drug delivery [90]. 

In addition, Hf(IV) possesses strong X-ray attenuation capability and can serve as a radio-sensitizer, including HfO_2_ nanocrystal assemblies [91], Hf(IV)-based nanoscale metalloorganic frameworks, and Hf carbon dots covering a photosensitizer used for combined radiation therapy and photodynamic therapy [92]. Many studies have explored the application of Hf-based nanomaterials in cancer imaging and diagnosis, exhibiting favorable safety profiles with controllable toxicity [93,94]. These nanomaterials not only hold the potential for tumor visualization and diagnosis but also for cancer therapy, due to their unique structures and chemical functionalities.

## 3. d-Elements of Group 5

### Vanadocenes and Other Vanadium Complexes

Many studies have established that V complexes reduce tumor growth and provide anticancer protection [95,96]. Vanadium complexes have been shown to exert either anti-proliferative or, in some cases, proliferative effects on various cell types, for instance, at low doses they stimulated, but at higher amounts inhibited, tumor formation. It has been supposed that the mechanism of anticancer activity of vanadium includes OS, DNA binding, cellular cycle regulation, and programed cellular death.

Vanadium is a biologically vital metal with the capability to adopt several oxidation states from +1 to +5 which provides V complexes unique characteristics and a crucial role in interactions with biomolecules. Particularly, V(IV) exerts functions in various biological systems by catalyzing reactive oxygen species (ROS) generation. Notably, V(IV) organometallic complexes with bis(cycopentadienyl) moieties or vanadocenes exhibit antitumor properties in vitro and in vivo, primarily through oxidative damage. It seems that the mechanisms of action of vanadocenes are dissimilar from those of titanocenes and other known metallocenes. Among the metallocenes, the most promising is vanadocene dichloride [95,96]. Its antitumor activity against human colon and lung carcinomas has been associated with the inhibition of DNA and RNA synthesis in tumors.

**Figure 9:**
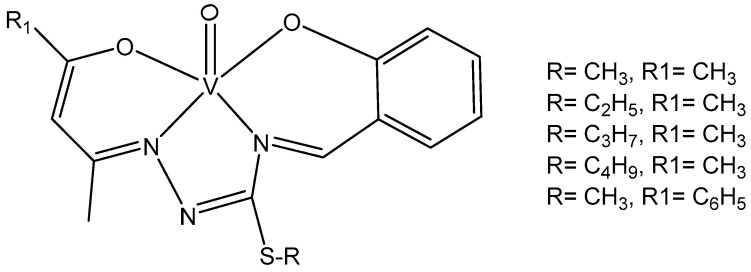
Oxovanadium(IV) complexes.

New oxovanadium(IV) complexes with acetyl- and benzoylacetone-S-alkyl-thiosemicarbazones (alkyl= methyl, ethyl, propyl or butyl) and salicylaldehyde ligands, Figure 9, have been recently synthesized [97]. The cytotoxicity of the obtained complexes has been determined against the MCF-7, MDA-MB-231, and 3T3 cell lines. All the complexes have shown improved cytotoxic effects than the drug 5-fluorouracil. The most effective was the complex holding the S-propyl group. Additionally, the capability of the tested compounds to inhibit xanthine oxidase, elastase, and neuraminidase has been studied and the complexes showed the inhibition of elastase and xanthine oxidase. The complexes containing ethyl and propyl groups have shown a better inhibition of neuraminidase than that of quercetin. 

Many biologically active reductants change the vanadate ion, [O_2_V]^+^, in coordination compounds, for instance, cis-[(OH)_2_O_2_V(V)]^−^, to the vanadyl ion, [OV(IV)]^2+^. The ion [OV(IV)]^2+^ can bind bioproteins and other cellular constituents with oxygen and nitrogen donors, where transferrin seems to be involved in this metabolism. Vanadate [O_2_V]^+^ and vanadyl [OV(IV)]^2+^ ions inhibit numerous phosphatases, ATPases, kinases, nucleases, etc. Vanadate(V) and vanadyl(IV) cations mimic the insulin effect, possibly by preventing the participation of protein phosphotyrosine phosphatase (PPTK). This leads to the inhibition of the insulin receptor kinase, responsible for the inactivation of insulin receptors, and to lipid peroxidation in hepatocytes. The inorganic salts [OV]SO_4_ and Na[VO_2_] inhibit the proliferation of cancer cells without affecting normal cells. The cytotoxic activity is improved in the presence of H_2_O_2_. [O-(maltolato)_2_V] has shown activity against MDAY-D2 implanted cancers [98]. Hydrogen peroxide dislocates oxo-groups from [O_2_V]^+^ compounds, thus forming side-bound peroxo-vanadates. Cytotoxicity against L1210 leukemia cells has been detected for peroxo-vanadium compounds, such as (NH_4_)_4_[O(O(O_2_)_2_V)_2_], M_3_[O(O_2_)_2_(C_2_O_4_)V], and NH_4_[O(O_2_)(malato)V], where M = K^+^, NH_4_^+^. In the presence of hydrogen peroxide, [OV(IV)]^2+^, which displays antitumor activity, cleaves DNA probably by producing hydroxyl ROS. In general, vanadium peroxidase activity appears to be dominant in its antineoplastic activity, perhaps via oxidative DNA damage; however, the inhibition of enzymes is also likely.

## 4. d-Elements of Group 6

### 4.1. Molybdenum(II) Complexes

Molybdenum is one of the ten essential biogenic metals [23,24,25,26,27,28,29], which is the only metal of the second transition series that is vital for life. The biochemical significance of Mo is due mostly to its various oxidation states, which afford easy and different electron-transfer routes. Its ability to form strong chemical bonds with O-, S-, and N-donor atoms is of great importance for the interactions with numerous biomolecules. Biological usages of Mo compounds are attributable to the ability of bioligands to chelate with trace Mo ions, to their specific mechanisms of action, and to their aptitude in generating ROS. These features can disturb the redox balance of biosystems leading to a rise in DNA damage, lipid peroxidation, cell toxicity, etc. In the literature, the molybdate anion MoO_4_^2−^ is reported to prevent lipid oxidation and defend antioxidant biological systems in vivo. The analogous tetra-thiomolybdate anion MoS_4_^2−^ is an appropriate Cu chelator not only for its ability to decrease Cu amounts, but also as a cytotoxic agent in breast cancer and esophageal carcinoma therapy in clinical trials [32]. Ammonium tetra-thiomolybdate is clinically approved as an anticancer agent. Furthermore, poly (lactic-co-glycolic) acid nanoparticles containing Mo octahedral clusters have been utilized for PDT of ovarian cancer [99].

**Figure 10:**
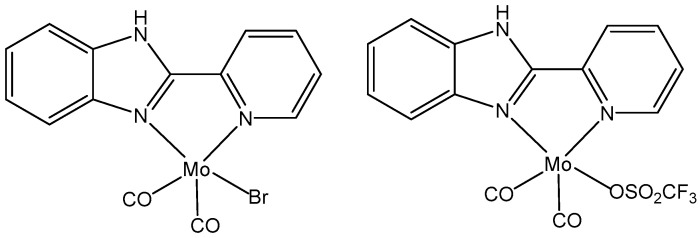
Molybdenum(II) complexes.

In the search for new antitumor metal complexes within the transition metals, the coordination compounds of molybdenum have been the least considered. Molybdenum(II) complexes with 2-(pyridine-2-yl)-1H-benzo[d]imidazole ligand, Figure 10, have shown cytotoxic effects against HeLa cells with IC_50_ values of 15 μM and 12 μM, respectively [100].

In therapeutic practice, molybdenum radioactive isotopes are utilized for diagnostic determinations for liver scanning and examination of blood circulation in muscles. The key molybdenum isotopes are ^95^Mo, ^96^Mo, ^98^Mo, and ^99^Mo, the last being the most commonly used diagnostic medical isotope [23,24,25,26,27,28,29,30].

### 4.2. Tungstenocenes

Inspired by the success of titanocene dichloride, molybdocene dichloride (Cp_2_MoCl_2_) and tungstenocene dichloride (Cp_2_WCl_2_) were developed as potential antitumor agents along with other metallocenes based on vanadium, niobium, and rhenium, capitalizing on their specific physicochemical properties [101]. The molybdenocenes and tungstenocenes bear a cis-dihalido motif like cisplatin, which is the primary reason for their interest in anticancer drug discovery. Molybdocene dichloride has received more attention than tungstenocene dichloride based on its more stable metal-Cp bonds, improved antineoplastic properties, and favorable hydrolysis chemistry. Tungstenocene chemistry was abandoned for many years in research, until the great cytotoxic activity of Cp_2_WCl_2_ derivatives bearing 3-hydroxy4-pyrone ligands, such as [(Cp_2_W(ethyl-maltolato)]Cl, was discovered and compared to the cytotoxicity of molybdenocenes [102]. The in vitro cytotoxic activity of these tungstenocenes, bearing (O,O–) donor ligands, has been improved, compared to Cp_2_WCl_2_, against HT29 colon cancer and MCF-7 breast cancer cell lines. 

**Figure 11:**
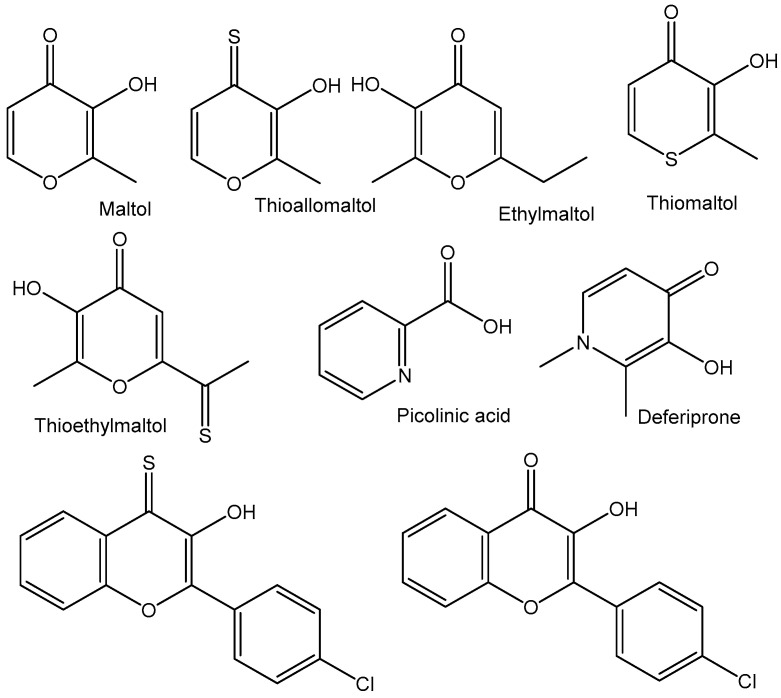
Ligands for tungstenocene complexes.

Furthermore, the structure modification by replacing chlorido ligands with bioactive chelating ligands has modulated the aqueous solubility, as well as stability, and the functionalization of the bidentate ligands provided has improved the antitumor potential. Another strategy for increasing anticancer activity could be the functionalization of the Cp ring with hydrophobic or hydrophilic groups and biologically active molecules. In fact, anticancer studies with tungstenocenes are very rare. Nevertheless, with the correct choice of chelating ligands (containing softer S-donors), an improved stability of the tungstenocenes can be attained, making this class of compounds more interesting in terms of anticancer research. A wide range of ligands have been utilized, including thiol derivatives of 3-hydroxy-4-pyrones, to improve the stability and the in vitro cytotoxicity. Recently, the synthesis, characterization, and biological studies of ten tungstenocenes containing (O,O–), (S,O–) or (N,O–) chelates, as well as mode-of-action studies, have been reported [103]. The bidentate ligand scaffolds have been coordinated to the cytotoxic tungstenocene dichloride, enhancing the cytotoxic activity and increasing the stability of the complexes in aqueous media. The influence on the aqueous stability of the W–S vs. W–O bonds and counter ion exchange from chloride to hexafluorophosphate has been discussed. The natural product 3-hydroxy-2-methyl-4-(1H)-pyrone (maltol), commonly applied as a ligand with a broad range of biologically relevant metals, has been used because of its potential bioavailability. Along with maltol, additional bioactive ligands such as thioallomaltol, ethylmaltol, thiomaltol, thioethylmaltol, picolinic acid, deferiprone, 3-hydroxy-2-(4′-chlorophenyl)-chromen-4-thione, and 3-hydroxy-2-(4′-chlorophenyl)-chromen-4-one (Figure 11) have been used. Ethylmaltol and its thioethylmaltol analogue were used for comparison purposes. Biologically active picolinic acid has demonstrated immunological, neuroprotective, and antiproliferative effects, while deferiprone has been shown to selectively kill tumor cells over normal cells. This group of ligands was suitable to determine the most promising chelating motif and to identify structure–activity relationships for the studied maltol-based complexes.

Complexes have been obtained by exchanging the chlorido ligands of the tungstenocene dichloride (Cp_2_WCl_2_) by the abovementioned ligands, resulting in positively charged complexes [Cp_2_W(L)]^+^. The cytotoxicity of the obtained compounds has been investigated by means of the MTT assay against the human cancer cell lines A549 (non-small cell lung carcinoma), SW480 (colon carcinoma), CH1/PA-1 (ovarian teratocarcinoma), and human lung fibroblasts IMR-90. Compounds with (thio)flavone ligands were the most active of this series. The picolinic acid and deferiprone derivatives have shown negligible activity, while the allomaltol, thioallomaltol and ethylmaltol derivatives have shown similar activity to the complex with maltol. Overall, the (S,O–) chelates have been more active than their (O,O–) counterparts with improved IC_50_ values. Tungstenocenes were found to be more active than molybdenocenes, which was probably due to their lower oxidation potentials and higher redox activity under physiological conditions compared with their Mo analogs. To find the mode of action of the new tungstenocenes, the potential to form ROS and apoptosis induction have been studied in HCT116, HT29, and MCF-7 cell lines. The most active complexes induced apoptosis and necrosis in a concentration-dependent manner in all three cell lines [103].

The biocompatibility and low toxicity of tungsten disulfide (WS_2_) nanotubes and nanoparticles make them suitable for biomedical applications. One potential application is photothermal therapy (PTT), a method for the targeted treatment of cancer, in which a light-responsive material is irradiated with a laser in the near-IR range. Polyoxometalates, a type of O-cluster anions formed by transition metals such as V, Nb, Ta, Mo, and W in their highest oxidation states, can implement a variety of sizes, shapes, and compositions. This adaptive nature allows their application in many medical fields [36]. Polyoxotungstates in medicine have been studied for their application as antitumor agents and for a range of other medicinal applications [37]. Nevertheless, they are not close to a clinical trial or a final application in the treatment of malignant diseases.

## 5. d-Elements of Group 7

### 5.1. Manganese(II) Complexes

**Figure 12:**
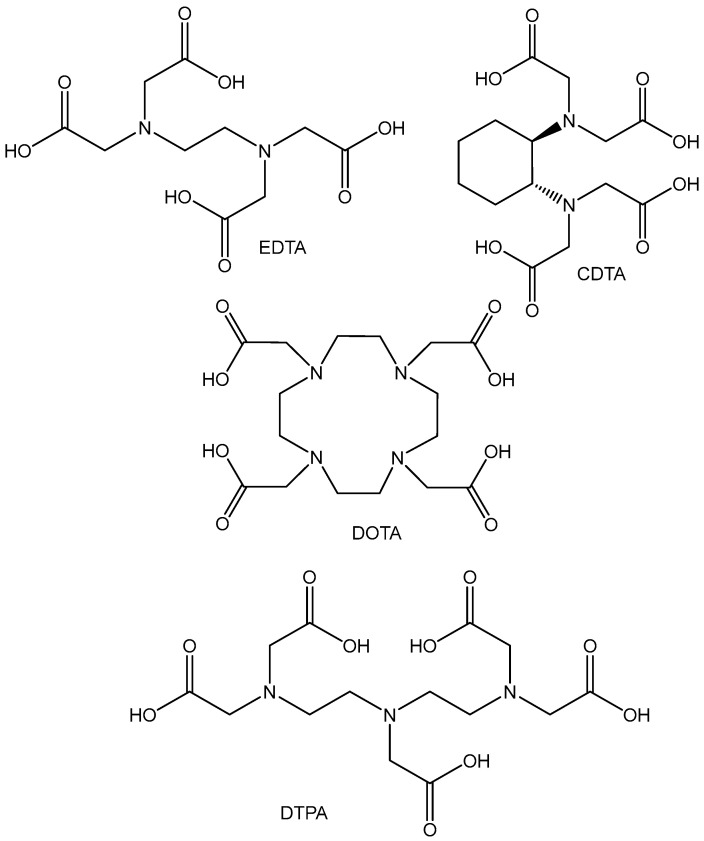
Structures of EDTA, CDTA, DOTA, and DTPA ligands.

The Mn(II) cation is characterized as a spherically polarized ion, which is a typical hard Lewis acid. That is why the most stable complexes of Mn(II) have been produced with hard ligands containing O and N donor atoms. Mn(II) cations coordinate with EDTA, CDTA, DOTA, DTPA, and their derivatives (Figure 12). Mn(II) ions possess five unpaired electrons, which leads to high paramagnetic moments of the complexes. Consequently, the obtained Mn(II) complexes with the above ligands have been studied as possible MRI contrast agents [46,47,48]. 

Manganese salts and complexes are investigational ambiguous cancerogenic agents. Although the antineoplastic properties of manganese complexes are underexplored, a number of cytotoxic Mn(II/III) complexes containing Schiff base, porphyrin, flavonoids, and polypyridyl ligands have been identified. Mn(II) complexes are generally stable and inert to biomolecules because of the protective effect of bioligands and can therefore alleviate the toxicity of Mn(II) cations. It has been proven that Mn(II) cations are mainly absorbed and transported by transferrin (Tf) and the transferrin receptor (TfR) system, which is highly expressed in cancer cells. The cellular mechanism of action of Mn(II/III) complexes is highly dependent on the nature of the coordinated ligand. Many studies have shown that manganese and its coordination compounds increase markers of OS in various cell lines because of intracellular ROS generation. Utilizing Mn over other more common endogenous metals like Cu and Fe leads to advantages in many physiologically important processes including Mn-mediated ROS generation. 

The antitumor Mn(II) complexes with thiosemicarbazone or hydrazone groups work by the induction of apoptotic cell death at relatively high concentrations [44]. Dithiocarbamate (R_2_NCS_2_^−^) ions strongly coordinate in a bidentate chelating mode metal cations in different oxidation states owing to the high electron density on the S atoms of the dithiocarbamato moieties. The use of low toxic dithiocarbamate ligands with different donor groups such as O and N in amino acids to synthesize complex compounds increases the anticancer activity [104]. 

**Figure 13:**
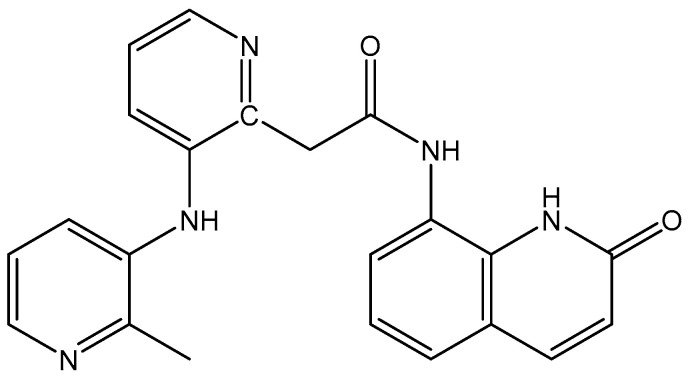
Structure of 2-di(picolyl)amine-N-(quinolone-8-yl)acetamide.

Wang et al. have reported two Mn(II) complexes [Mn(QA)Cl_2_] and [Mn(QA)(OAc)(H_2_O)_2_]OAc where OAc = acetate ion and QA = 2-di(picolyl)amine-N-(quinolone-8-yl)acetamide (Figure 13) [105]. The obtained manganese(II) complexes were tested against the HepG-2 cell line. The IC_50_ values revealed that [Mn(QA)Cl_2_] was more cytotoxic than the other complex towards the HepG-2 cell line.

**Figure 14:**
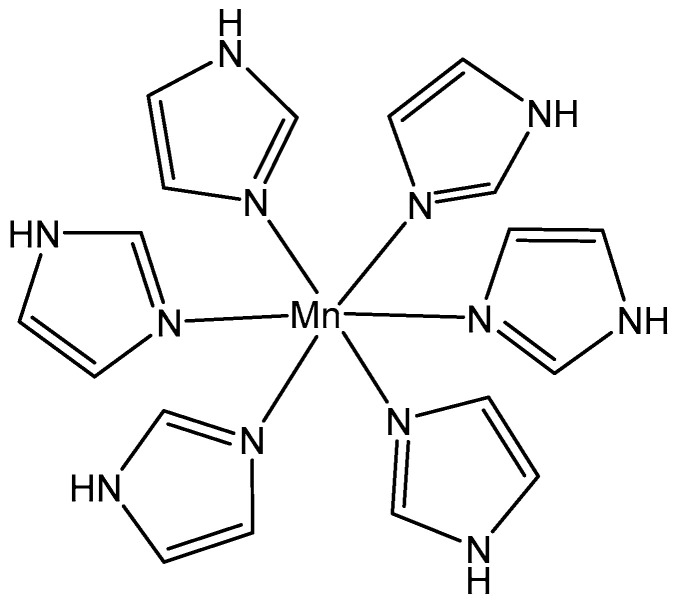
Mn(II) complex of imidazole.

A new Mn(II) complex has been derived from the condensation of 2-aminophenol with 2-hydroxynapthaldehyde [106]. The in vitro cytotoxicity of the synthesized complex has been screened out against HCT-116, HepG-2, and MCF-7 cancer cell lines, showing better cytotoxicity against MCF-7, followed by HepG-2 and HCT-116 cancer cell lines. Khan et al. have recently synthesized a Mn(II) complex of imidazole, Figure 14 [107]. The MTT assay indicated that this complex can be used as anticancer agent against the RAW 264.7 cell line. 

**Figure 15:**
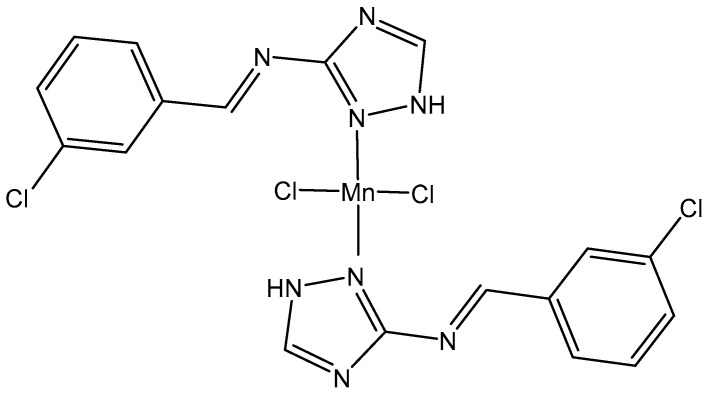
Chemical structure of a Mn(II) complex with N-(-(3-chlorobenzylidene)-1H-1,2,4-triazol-3-amine.

The Manganese(II) complex of N-(3-chlorobenzylidene)-1H-1,2,4-triazol-3-amine, Figure 15, has been investigated and found to exert anticancer properties against the human breast cancer MCF7 cell line [108].

**Figure 16:**
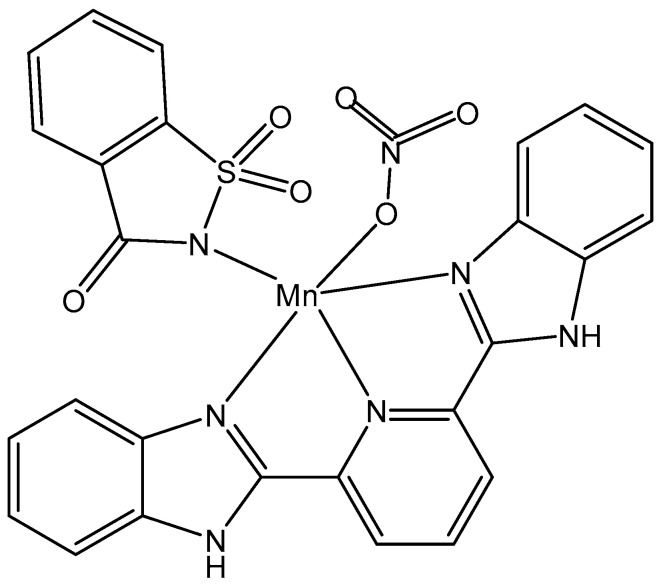
Mn(II) complex with saccharin and 2,6-bis(2-benzimidazolyl)pyridine.

The Mn(II) coordination compound of the ligands saccharin and 2,6-bis(2-benzimidazolyl)pyridine, Figure 16, displayed around a 3-fold stronger cytotoxicity towards A549 and HT-29 tumor cells than cisplatin with an IC_50_ = 7.86 μM and 3.50 μM, respectively. The compound has shown no toxic effects against normal MCF10A cells. The Mn(II) complex has induced cell arrest in the G0/G1 phase, enhanced intracellular ROS amounts, produced mitochondrial dysfunction, and shown nuclease activity [109]. 

**Figure 17:**
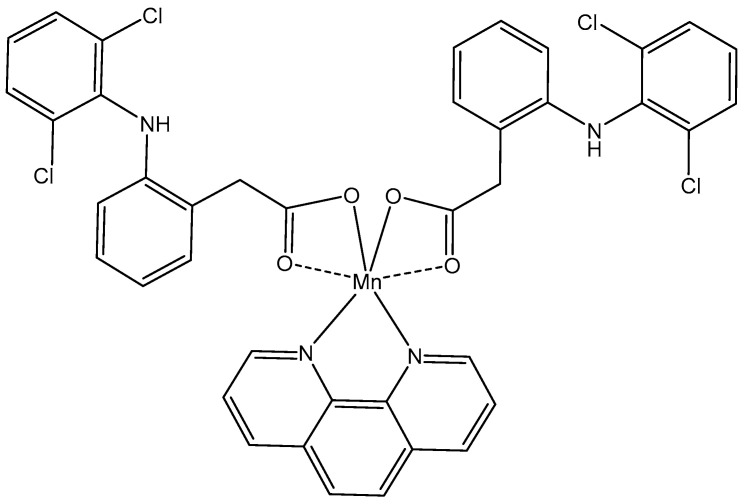
Mn(II) complex of 1,10-phenanthroline.

The Mn(II) complex containing 1,10-phenanthroline and two diclofenac molecules (common non-steroidal inflammatory drug (*NSAID*) type ligands), Figure 17, has demonstrated a high antiproliferative activity against breast (HMLER) and breast CSC-enriched (HMLERshEcad) cancer cell lines (IC_50_ = 0.186 μM and 0.137 μM, respectively). Notably, the complex showed around 10–40-fold better activity than cisplatin or salinomycin and above 60-fold higher potency against breast CSCs cells than against normal skin fibroblasts. Its mechanism of action includes intracellular ROS generation and cyclooxygenase-2 (COX-2) inhibition [110].

The manganese(II) cation possesses many characteristics that are valuable for MR, PET, and positron emission tomography–magnetic resonance imaging (*PET*–*MRI*), such as kinetic lability, a long relaxation time, high spin number, and fast water exchange. The Mn(II) ion is one of the most promising options compared to the Gd(III) ion as a result of its lower thermodynamic stability and kinetic inertness than that of Gd(III) or other active metal ions. This is because of the smaller charge of Mn(II) and the minimum ligand-field stabilization energy due to its d^5^ electron configuration [44].

Several manganese radionuclides are currently known with their valuable properties; for example, Mn-51, Mn-52m, and Mn-52g are positron emitters used in MR, PET, and PET/MR imaging [45]. ^51^Mn (T_1/2_ = 46 min) has a satisfactory β^+^ branching fraction similar to that of ^68^Ga (T_1/2_ = 68 min), which is appropriate for the imaging of fast bioprocesses. The β^+^ energy of ^52m^Mn is higher than that of ^51^Mn. In contrast to ^51^Mn and ^52m^Mn isotopes, the most promising ^52g^Mn has a suitable long half-life (T_1/2_ = 5.6 d), which is advantageous for target separations and chemical handling of the radionuclide. Furthermore, its half-life is well suited for the investigation of slow bioprocesses, for instance, the pharmacokinetics of antibodies. For medical imaging, the attention is on the long-living ^52g^Mn and its effective usage for radiolabeling of molecules, the imaging of Mn-dependent biological processes, and the development of PET/MRI probes in combination with the paramagnetic contrast agent ^nat^Mn. Since ^nat^Mn(II) is paramagnetic and PET isotopes of the metal are available, isotopically radiolabeled Mn-based PET/MRI contrast agents could be interesting agents with a high potential to become new emerging radiometals for nuclear medicine applications. The main challenge is the possibility to find suitable chelators, which can yield Mn(II) complexes of tolerable stability and relaxivity. Various approaches towards the development of these Mn(II) complexes have been broadly described in the literature [45]. 

### 5.2. Technetium Complexes

**Figure 18:**
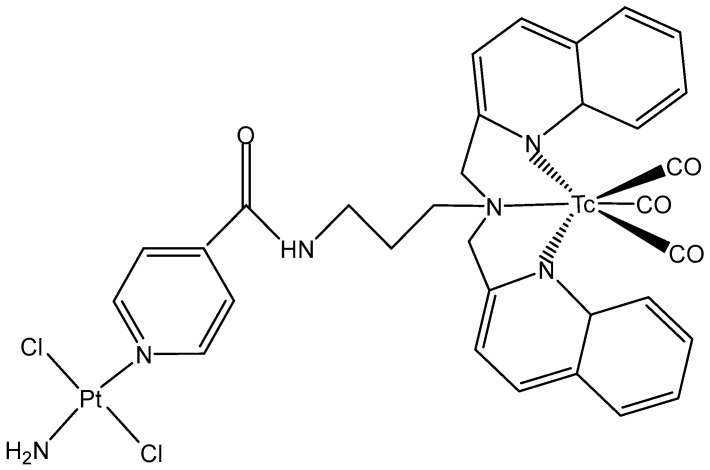
Molecular structure of a ^99m^Tc-Pt heterometallic complex.

As members of Group 7 of the periodic table, the elements technetium and rhenium possess a rich coordination chemistry. In the heterometallic complex (Figure 18), the ^99m^Tc-tricarbonyl unit has been combined with a Pt complex to allow biodistribution investigations and imaging studies in normal mice. The intensity of radioactivity has been found to be higher in the excretory liver and kidney, where the complex accumulated the most [111]. 

Technetium is a radioactive element, with no stable isotopes. Technetium-99m is the most extensively used radioisotope in nuclear diagnostic imaging. Its short half-life (T_1/2_ = 6 h), easy assimilation into a variety of carrier molecules, low energy γ-emission, and fast excretion make it perfect for obtaining images of the most important internal organs and skeleton of the human body. Technetium-99m is easily generated at the bedside from the long-lived radioisotope molybdenum-99. Many ^99m^Tc radiopharmaceutical drugs, mainly coordination complexes, are currently in use for the imaging of different body organs, including bones, brain, lungs, thyroid gland, myocardium, liver, etc., [49,50]. Modern trends in the radiopharmaceutical chemistry of technetium focus on the ‘labeling’ of biologically active molecules such as peptides, steroids, or other receptor-seeking units. The bioligands play a central role in the targeting functions of the complexes, for instance, phosphate and phosphonate complexes for bones. Bone scanning by means of ^99m^Tc methylene diphosphonate (^99m^Tc-MDP) is used to estimate renal osteodystrophy. Methylene diphosphonate adsorbs to bones and shows a better affinity to locations of new bone formation [49,51]. SPECT with ^99m^Tc-glucoheptonate (^99m^Tc-GHA) has shown promising results in brain scans for the differentiation of recurrent brain tumors from radiation necrosis. Cardiolite (^99m^Tc-sestamibi) and Neurite (^99m^Tc-disicate) have been used for folate-receptor-positive tumor tissues. ^99m^Tc-MIP-1404 is used for prostate cancer imaging [49,52]. 

### 5.3. Rhenium Complexes

**Figure 19:**
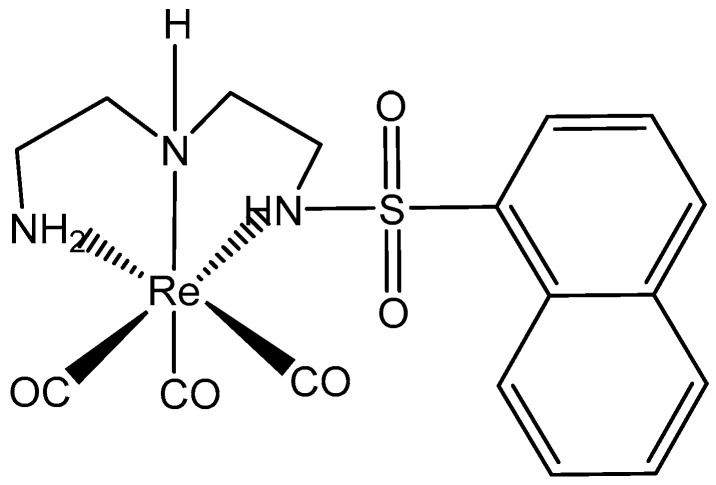
Re(I) tricarbonyl complex, containing 1-naphthalenesulfonamide.

Of the variety of metal cations being studied for therapeutic applications, rhenium and its complexes are among the most promising antitumor agents. Perrhenate ion ReO_4_^−^ is one of the least toxic transition metal ions; therefore, Re compounds are very attractive in anticancer drug discovery. The Re(I) tricarbonyl complex compound, Figure 19, holding 1-naphthalenesulfonamide has been reported to have more activity than cisplatin against NCI-H292 lung cancer cells, with IC_50_ = 9.91 μM. This compound was around 5-fold less toxic against normal lung fibroblast cells. In contrast, a similar 2-naphtalenesulfonamide complex had no cytotoxic activity against the NCI-H292 cell line [112]. 

**Figure 20:**
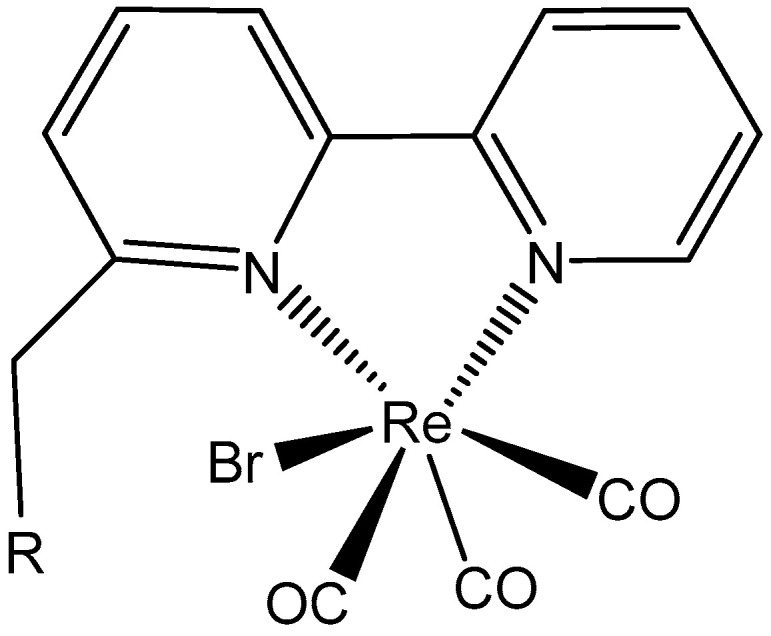
Re(I) tricarbonyl complexes containing bipyridine derivatives.

Re(I) tricarbonyl complexes containing bipyridine derivatives with different substituents, Figure 20, have exhibited strong cytotoxic effects towards HCT-116 cells (IC_50_ = 5.0–6.0 μM) and are less toxic towards normal fibroblast cells. Re(I) tricarbonyl complexes with bipyridine ligands have not caused cardiotoxic, hepatotoxic, or myelosuppressive effects and have been shown to possess stronger anticancer and anti-angiogenic activity than the well-known drugs cisplatin or sunitinib. Re(I) complexes containing diethylamine or diisopropylamine instead of diisobutylamine were not selective towards HCT-116 cells in vitro, while derivatives disubstituted with 2-toluidine or trimethylamine possessed around ten times less antitumor activity towards HCT-116 cells [113].

**Figure 21:**
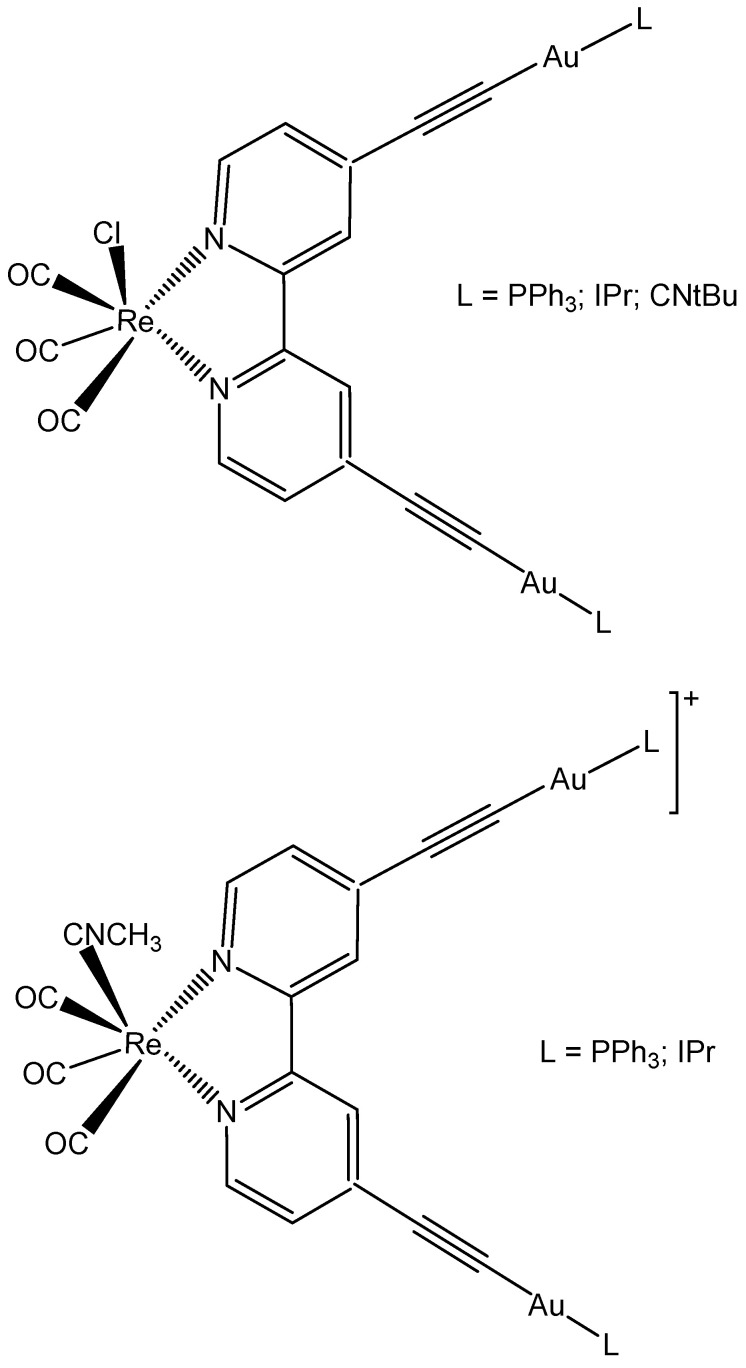
Neutral and cationic heterotrimetallic complexes.

A series of Re(I)/Au(I) heterotrimetallic complexes with the general formula *fac*-[Re(CO)_3_(bipy(CC)_2_-(AuL)_2_)X]*^n^*, where bipy(CC)_2_ = 4,4’-alkynyl-2,2’-bipyridine and L = triphenylphosphine (PPh_3_); [1,3-bis(2,6-diisopropylphenyl)-imidazol-2-ylidene] (IPr); *tert*-butyl isocyanide (CNtBu) and X = Cl^−^ (*n* = 0); CH_3_CN (*n* = 1), (Figure 21), have been obtained and compared with *fac*-[Re(CO)_3_(bipy(CC)_2_)X]*^n^*. The cytotoxic activity of gold(I)-rhenium(I) heterometallic neutral and ionic complexes has been assessed against A549 and He-La cell lines and the results have shown better selectivity towards HeLa cells, probably associated with the Au fragment [114]. The cationic complexes have generated better results than the neutral complex compounds. The cellular uptake of gold(I) ancillary ligands follows the order -PPh3>-CNtBu>-Ipr. In the case of the A549 cell line, all the complexes have accumulated outside the cells, whereas in the HeLa cell line, the cationic complexes have been found very close to the membrane, signifying some kind of interaction of the compounds with the cellular membrane, thus elucidating the observed cellular selectivity demonstrated in A549 and He-La cell lines. The stronger cytotoxicity of *fac*-[Re(CO)_3_(bipy(CC)_2_)X]*^n^* against HeLa cells can be connected with its solubility. 

**Figure 22:**
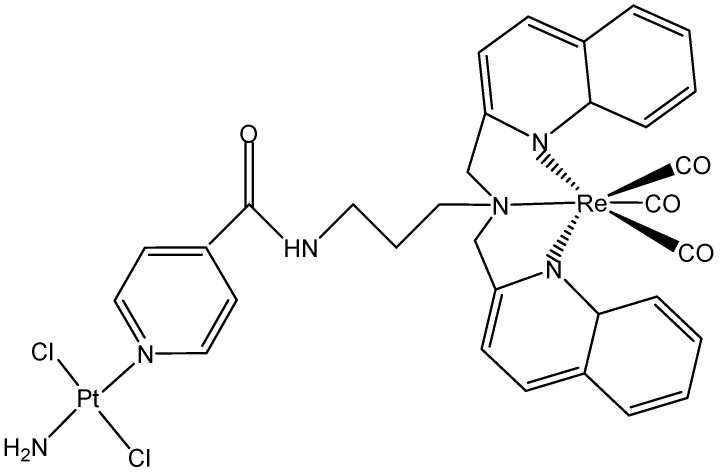
Molecular structure of the Re–Pt heterometallic complex.

The combination of two different metal centers, Pt(II) with therapeutic properties and Re(I) with diagnostic properties for theranostics, has been investigated [111]. The new heterometallic complex (Figure 22) has a Re moiety with photosensitizing properties and a non-classical Pt center with basic cytotoxicity in the dark.

**Figure 23:**
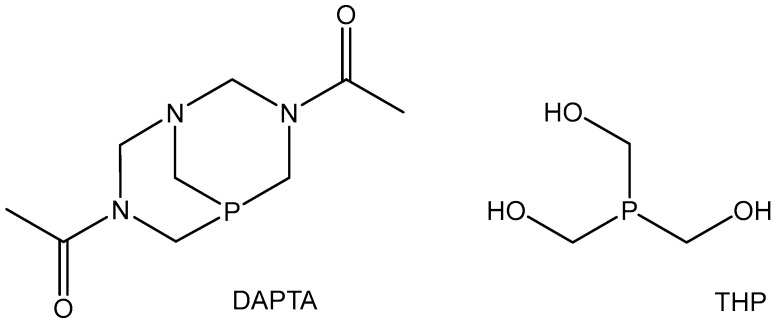
Phosphines 3,7-Diacetyl-1,3,7-triaza-5-phosphabicyclo[3.3.1]nonane (DAPTA) and tris(hydroxymethyl)phosphine (THP).

There is great interest in the application of rhenium in medical diagnostics for imaging determinations based on its radioactive beta-emitter isotopes ^186^Re (T_1/2_ = 16.9 h) and ^188^Re (T_1/2_ = 89.2 h) that combine diagnosis by radio imaging with conventional γ-cameras with therapeutic actions, allowing an effective energy transfer to cancer tissue. Radioisotopes of rhenium, ^188^Re (β-emitter) and ^186^Re with decays by electron capture and beta-emission, are used in the treatment of malignant cancers, bone metastases, rheumatoid arthritis, etc. Colloidal S-particles labelled with rhenium-186 are used in radiosynovectomy in rheumatoid arthritis therapy and ^188^Re-1,1-hydroxyethylidenediphosphonate (^188^Re-HEDP) for bone pain palliation in cases of prostate cancer [55]. Rhenium radioisotopes have both particle emissions with characteristics appropriate for targeted treatment and photon emissions, which may be used for diagnostic investigation purposes by SPECT. The best studied coordination complexes of technetium and rhenium are those with carbonyl and nitrosyl ligands such as [M(CO)_3_]^+^, [M(CO)_2_(NO)]^2+^, which is isoelectronic to [M(CO)_3_]^+^, as well as [ReCl_2_(CO)_2_(NO)]_2_ and [M(CO)_3_X_3_]^2−^ anions (M = Re, Tc; X = Cl, Br). It is clear from the structures that for both metals, substitution of more than one CO ligand was not observed. Many of the Re(I) tricarbonyl complexes were noted as photosensitizers for PDT [115]. New Re(I) tricarbonyl complexes containing the water-soluble phosphines 3,7-Diacetyl-1,3,7-triaza-5-phosphabicyclo[3.3.1]nonane (DAPTA) and tris(hydroxymethyl)phosphine (THP), Figure 23, have been found not to be toxic for HeLa, A2780, and A2780*cis*R cell lines in the dark (IC_50_ > 200 μM). However, after 365 nm light irradiation clear phototoxicity of the compounds towards HeLa, A2780, and A2780*cis*R cells has been observed with low values of IC_50_. The mode of action possibly includes the release of CO and ROS [116]. Further studies with biologically relevant organic ligand systems and chelators (viz., cyclic thioethers, diimines, aminocarboxylic acids, N-heterocyclic carbenes, phenylimido, etc.), which are promising for the bifunctional approach, are currently in progress and it is to be expected that this new class of technetium and rhenium compounds will play a role in future radiopharmaceutical research.

## 6. Conclusions and Prospective

In this review, the rapid progress in the design and application of metal-based compounds (metallocenes and metal complexes) of elements from Groups 4 to 7 in cancer treatment and diagnostics has been summarized. Cancer imaging, diagnostics, and therapies that include metal-based compounds have improved in the last several years. The transition metal complexes and metallocenes of the reviewed elements possess a good anticarcinogenic activity. Some of them have proved to be very active antiproliferative agents against various cancer cell lines. The results showed that the complexes increase the cytotoxic activity with respect to the free ligands. Among numerous metal-based compounds reported, some candidates with these elements have been found as good alternatives to classical anticancer drugs, offering lower toxicity, broader ranges of oxidation states, slow ligand exchange rates, and various coordination numbers and geometries, which provide different modes of action and reactivity. The possible application of transition metal complexes of the studied groups of the periodic table is an underdeveloped research area and is full of opportunities for further progress.

## Figures and Tables

**Table 1:** Biological functions, medical anticancer applications, and toxic effects of metals of Groups 4 to 7 and their compounds.

Element	Location and Biofunctions	Compounds with Anticancer Activity	Toxicity, Antidotes	References
Titanium	Bio-stimulant; one of the most biocompatible metal implants	Ti(IV) complexes—for treatment of cancer (budotitane, Ti–salan, and titanocenes)	Ti metal is not toxic; TiO_2_ is a carcinogen	[11,12]
Vanadium	Stabilizes blood sugar levels; protects bones and teeth; insulin mimetic	Vanadocenes—inhibition of cancerous tumor growth; insulin-mimetic agents	Non-serious hazard; V_2_O_5_ is more toxic	[13,14,15,16,17,18,19]
Tantalum	Biocompatible and non-reactive with bio-tissues	Ta metal in long-term surgical implants and bone defects repairing	Low-soluble Ta compounds are moderately toxic	[20,21]
Molybdenum	Part of enzyme xanthine oxidase, in purine metabolism	MoO_4_^2−^ prevents oxidation of lipids; protects antioxidant systems; MoS_4_^2−^-Cu chelator, in breast cancer and esophageal carcinoma; isotopes—for radio-diagnostics	Excess of Mo disturbs purine metabolism—endemic gout	[22,23,24,25,26,27,28,29,30,31,32]
Tungsten	Essential for some anaerobic bacteria	Polyoxotungstates—antiviral, antibacterial, anticancer agents	W quantities in nature are low; W is nontoxic	[33,34,35,36,37]
Manganese	Bone, liver, lungs, muscles, pancreas, kidney; Mn-SOD in mitochondria	Mn—part of enzymes catalyzing redox reactions; PET and PET/MR imaging	Excess of Mn causes manganism; in ROS production	[38,39,40,41,42,43,44,45,46,47,48]
Technetium	Tc—in radiation imaging as a tracer; ^99m^Tc concentrates in gastrointestinal tract and thyroid gland	^99m^Tc (γ-emitter)—in SPECT for diagnostic imaging of bone, brain, lungs, thyroid, liver; radiation treatment of cancers with minimal adverse effects	Short half-life and rapid excretion of Tc radioisotopes minimize the toxic effects	[49,50,51,52]
Rhenium	β-emitters ^186^Re and ^188^Re in radio imaging; therapeutic properties on malignant tumors, bone metastases, rheumatoid arthritis	^188^Re-HEDP for bone pain palliation in prostate cancer, ^188^Re-P2045—for therapy of small cell lung cancer and neuroendocrine carcinomas; Re(I) complexes—anticancer properties and reduce ROS production	No reports on the toxicity of metal and its compounds	[53,54,55]
